# Supplementary material for: SynDLP is a dynamin-like protein of Synechocystis sp. PCC 6803 with eukaryotic features
Source: Nat Commun. 2023 Apr 14;14:2156. doi: 10.1038/s41467-023-37746-9 (PMC10104851; doi:10.1038/s41467-023-37746-9)
Supplement: Supplementary file 1 — Supplementary Information [file 41467_2023_37746_MOESM1_ESM.pdf]

Supplementary Information

*Syn*DLP is a dynamin-like protein of *Synechocystis* sp. PCC 6803 with eukaryotic features

Lucas Gewehr[1,†], Benedikt Junglas[2,3,†], Ruven Jilly[1], Johannes Franz[4], Wenyu Eva Zhu[1], Tobias Weidner[5], Mischa Bonn[4], Carsten Sachse[2,3,6*] and Dirk Schneider[1,7*]

[1] Department of Chemistry, Biochemistry, Johannes Gutenberg University Mainz, Germany.

[2] Ernst Ruska-Centre for Microscopy and Spectroscopy with Electrons (ER-C-3): Structural Biology, Jülich, Germany.

[3] Institute for Biological Information Processing (IBI-6): Cellular Structural Biology, Jülich, Germany.

[4] Max Planck Institute for Polymer Research, Ackermannweg 10, 55128 Mainz, Germany.

[5] Department of Chemistry, Aarhus University, Langelandsgade 140, 8000 Aarhus C, Denmark

[6] Department of Biology, Heinrich Heine University, Universitätsstr. 1, Düsseldorf, Germany.

[7] Institute of Molecular Physiology, Johannes Gutenberg University Mainz, Germany.

*To whom correspondence should be addressed: c.sachse@fz-juelich.de; Dirk.Schneider@uni-mainz.de

† These authors contributed equally to this work

Supplementary Table 1: Primers used in this study. F = forward primer, R = reverse primer

**Primer**	**Used for**	**Sequence (5' → 3')**
**F_NsiI_slr0869**	Amplification of *slr0869* with NsiI restriction site	GGCCATGCATATGTCCAAGATTGCGCCCCA
**R_XhoI_slr0869**	Amplification of *slr0869* with XhoI restriction site	GGCCCTCGAGTTCTACTATTTCCACAAAAT
**F_TG2329GC**	Site-directed mutagenesis C777A	CGAGTATTGTTAGCATTAATGAAGCTTTAAAAGCCATGCAAATTTTTG
**R_TG2329GC**	Site-directed mutagenesis C777A	CAAAAATTTGCATGGCTTTTAAAGCTTCATTAATGCTAACAATACTCG
**F_pET303-*Syn*DLP-HPRN552-555AAAA_insert**	Mutation of HPRN552-555AAAA via Gibson assembly, insert PCR	CTTTAAGAAGGAGGTCTAGAATGCATATGTCCAAGATTGCGCCCCAATG
**R_pET303-*Syn*DLP-HPRN552-555AAAA_insert**	Mutation of HPRN552-555AAAA via Gibson assembly, insert PCR	AAGGAGCTGTGGACGCTGCGGCCGCACTTTCCGTTGCTCGCTTATAGGC
**F_pET303-*Syn*DLP-HPRN552-555AAAA_vector**	Mutation of HPRN552-555AAAA via Gibson assembly, vector PCR	GCGGCCGCAGCGTCCACAGCTCCTTTTATTGCAGTTTTG
**R_pET303-*Syn*DLP-HPRN552-555AAAA_vector**	Mutation of HPRN552-555AAAA via Gibson assembly, vector PCR	CATTGGGGCGCAATCTTGGACATATGCATTCTAGACCTCCTTCTTAAAG
**F_slr0869_upstream**	Checking the *slr0869* knock-out *in vivo*	TATCAAGCTTGGGAGTGGCACAAGAAACAA
**R_slr0869_downstream**	Checking the *slr0869* knock-out *in vivo*	TAGTGGATCCTGCTTGTATGTCAGAAAGGT
**F_AA181GC**	Site-directed mutagenesis K61A	GGCTTTTAGTGCCGGTGCGTCCATGCTGATCAATG
**R_AA181GC**	Site-directed mutagenesis K61A	CATTGATCAGCATGGACGCACCGGCACTAAAAGCC
**F_pET303-*Syn*DLP-648-665GS_insert**	Mutation of P648 – L665 to a GS-linker via Gibson assembly, insert PCR	CTTTAAGAAGGAGGTCTAGAATGCATATGTCCAAGATTGCGCCCCAATG
**R_pET303-*Syn*DLP-648-665GS_insert**	Mutation of P648 – L665 to a GS-linker via Gibson assembly, insert PCR	AGAACCCGACCCGGAACCGGAGCCAGAACCGGAACCTGACCCTGAACCCGAGCCACTTTCCCGCACATAACGATCAC
**F_pET303-*Syn*DLP-648-665GS_vector**	Mutation of P648 – L665 to a GS-linker via Gibson assembly, vector PCR	TGGCTCCGGTTCCGGGTCGGGTTCTCAGCAAACTTCCCAGGG

**R_pET303-*Syn*DLP-648-665GS_vector**	Mutation of P648 – L665 to a GS-linker via Gibson assembly, vector PCR	CATTGGGGCGCAATCTTGGACATATGCATTCTAGACCTCCTTCTTAAAG
**F_pET303-*Syn*DLP-667-675GS_insert**	Mutation of Q667 – Q675 to a GS-linker via Gibson assembly, insert PCR	CTTTAAGAAGGAGGTCTAGAATGCATATGTCCAAGATTGCGCCCCAATG
**R_pET303-*Syn*DLP-667-675GS_insert**	Mutation of Q667 – Q675 to a GS-linker via Gibson assembly, insert PCR	ACCAGAGCCTGATCCCGAACCCGAGCCCTGGAGAGTTTGCCGGAATTGATAAATGG
**F_pET303-*Syn*DLP-667-675GS_vector**	Mutation of Q667 – Q675 to a GS-linker via Gibson assembly, vector PCR	CTCGGGTTCGGGATCAGGCTCTGGTGCGATCGTGGAAGCG
**R_pET303-*Syn*DLP-667-675GS_vector**	Mutation of Q667 – Q675 to a GS-linker via Gibson assembly, vector PCR	CATTGGGGCGCAATCTTGGACATATGCATTCTAGACCTCCTTCTTAAAG

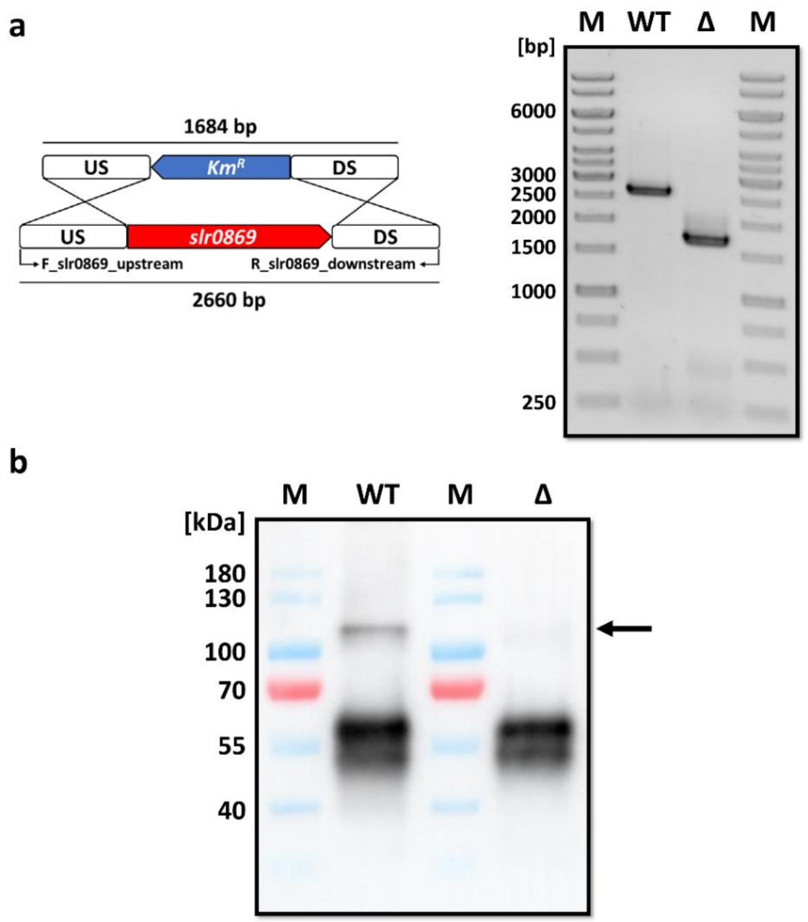

Supplementary Figure 1: *Syn*DLP expression *in vivo*.

(a) A *Syn*DLP knock-out strain (Δ) was generated via homologous recombination using a plasmid containing flanking regions up- and downstream (US and DS in the scheme) of the gene coding for *Syn*DLP (*slr0869*) and a kanamycin resistance cassette (Km^R) between the flanking regions. For selection, the strain was grown in BG11 medium[1] containing up to 100 µg/ml kanamycin. After phenolic DNA extraction of the strains, complete gene deletion was verified by PCR using the primer pair F_slr0869_upstrean and R_slr0869_downstream (Supplementary Table 1), followed by restriction digestion of the template DNA using the restriction enzyme DpnI and subsequent separation of the PCR products via agarose gel electrophoresis. DNA containing bands were stained using ethidium bromide. The expected PCR product size for the wt is 2660 bp and for the *slr0869* knocked-out strain 1684 bp, respectively. M = marker. Representative agarose gel of three independent experiments showing the same results. As the *slr0869* gene could be completely deleted, the native *Syn*DLP protein appears to be non-essential for the cyanobacterium, at least under the chosen growth conditions. The deletion strain grows like the wt and did not show severe defects in photosynthetic performance. Thus, our initial *in vivo* experiments indicated no altered phenotype of the *Syn*DLP knock-out strain, at least under standard growth conditions. (b) 100 ml of *Synechocystis* sp. PCC 6803 WT culture and the *Syn*DLP knock-out strain were grown in a shaking incubator at 130 rpm, 30°C and illuminated with 30 µE to an OD_{750} = 1.4. After cell disruption, *Syn*DLP was immunoprecipitated using Sepharose-Protein A beads and an antibody raised in a rabbit against part of *Syn*DLP (α-*Syn*DLP; dilution: 1:2000; Davids Biotechnologie, Regensburg, Germany). The truncated *Syn*DLP variant used for antibody production consisted of aa 300 – 812 to omit the conserved GD residues in the nucleotide-binding site (Fig. 1h) and, thus, increase the specificity of the antibody. The immunoprecipitated proteins were separated via SDS-PAGE and *Syn*DLP was visualized by Western Blot analysis using α-*Syn*DLP as primary and α-Rabbit-HRP conjugate (Dilution: 1:160000; Sigma-Aldrich Chemie GmbH, Taufkirchen, Germany) as secondary antibody. The height of a *Syn*DLP control band is marked by an arrow. The lower bands at ca. 55 kDa resulted from cross-reactions of the antibodies used for immunoprecipitation and Western Blot. M = marker. Representative Western Blot of two independent experiments showing the same results.

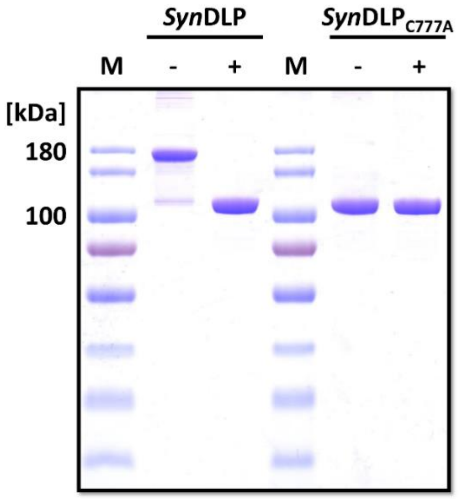

Supplementary Figure 2: The C777A mutation abolishes the formation of the intramolecular disulfide bridge.

Purified *Syn*DLP and *Syn*DLP$_{C777A}$ were analyzed via SDS-PAGE under reducing (100 mM DTT, lane +) and non-reducing (0.1 mM DTT, lane -) conditions. M = marker. Representative gel of three independent experiments showing the same results.

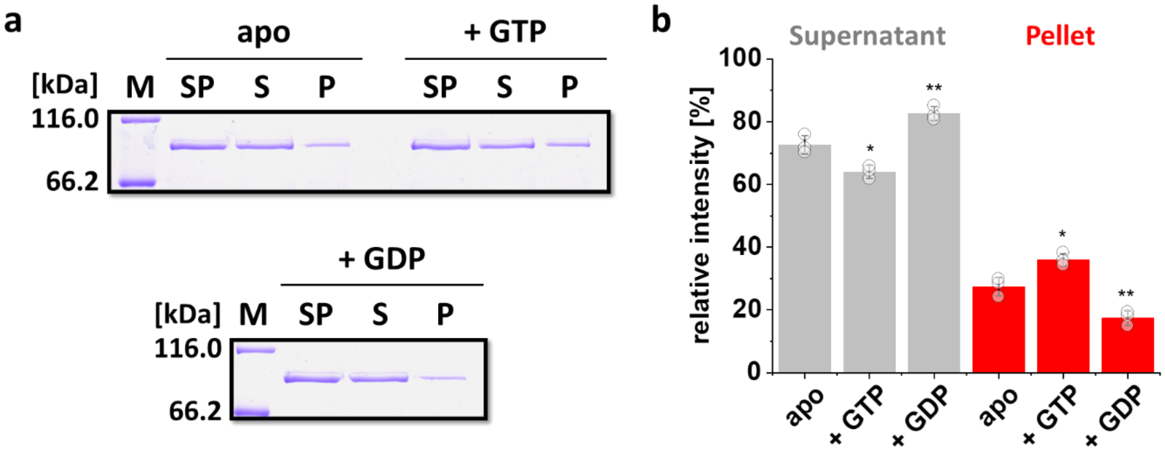

Supplementary Figure 3: Oligomerization of *Syn*DLP in the presence of nucleotides.

A sedimentation assay was performed to estimate the oligomerization behavior of *Syn*DLP in the presence *vs*. absence of nucleotides. 0.5 µM *Syn*DLP were incubated with or without 2 mM GTP/GDP in reaction buffer at 4°C for 30 min. One part of the reaction was removed before ultracentrifugation to receive the combined sample of supernatant and pellet (SP). After ultracentrifugation (60000g, 30 min, 4°C), the supernatant (S) was subtracted, and the pellet (P) was resuspended in an equal volume of SDS sample buffer. All samples were transferred into SDS sample buffer, boiled at 95°C for 5 min, and separated via SDS-PAGE. M = marker. (a) A representative gel showing the distribution of *Syn*DLP into S and P fractions dependent on the added nucleotide. (b) SDS-PAGE gel band intensities were determined using the software Fiji-ImageJ[2]. Grey bars show the relative band intensities of the supernatant and red bars those of the pellet. Mean of independent experiments (n=3) and error bars (S.D.) are shown. Single measurements are shown as circles. ns = not significant ($P > 0.05$), * $P < 0.05$, ** $P < 0.01$, *** $P < 0.001$ based on a two-sided unpaired Student's t-test. Either the GTP ($P = 0.014$) or the GDP ($P = 0.0096$) bound state are compared to the respective apo state.

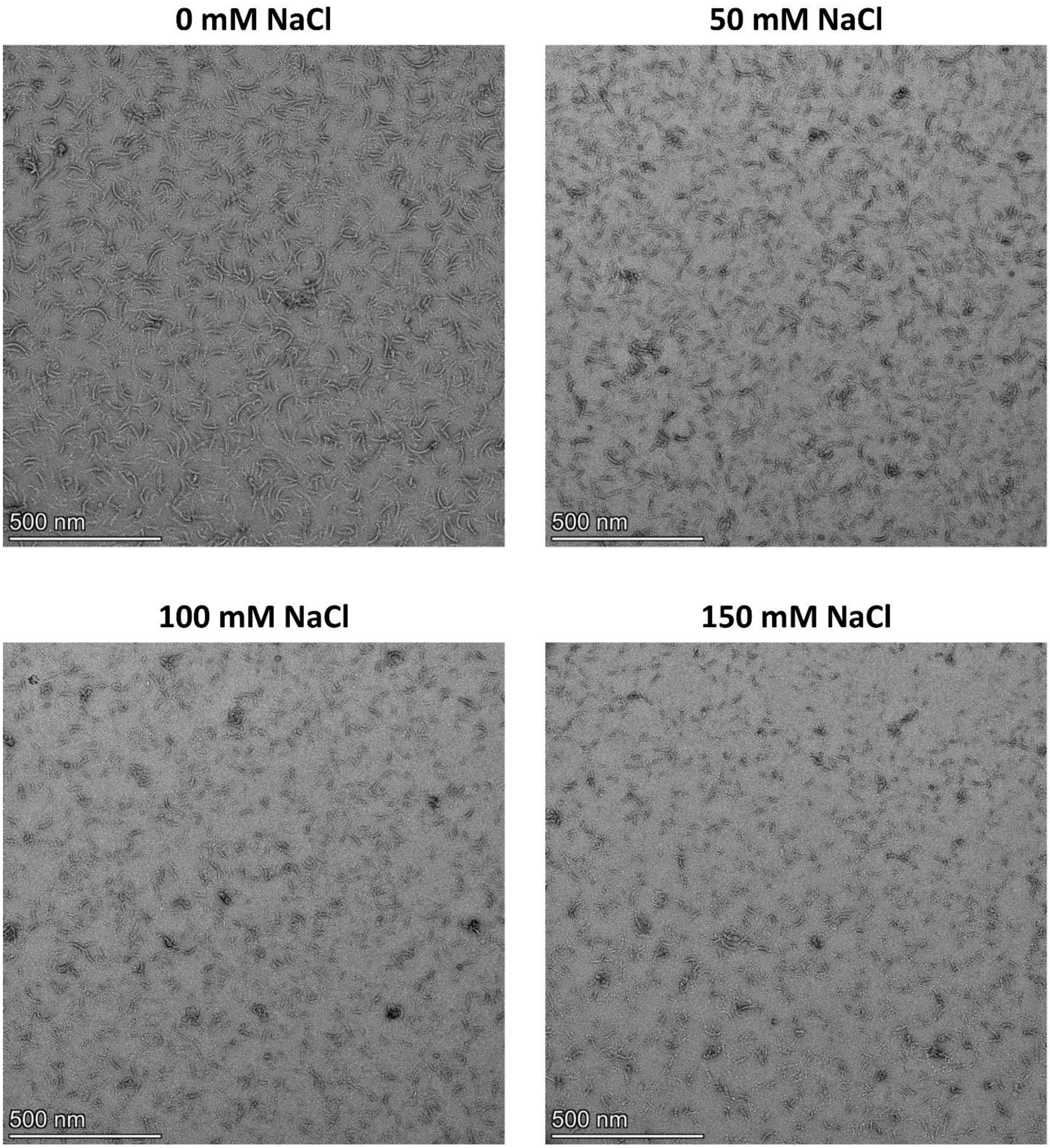

Supplementary Figure 4: Effect of increasing NaCl concentration on shape and size of *Syn*DLP oligomers.

Negative stain EM micrographs of 10 µM *Syn*DLP in 20 mM HEPES pH 7.4, 7.5 mM KCl, 5 mM $MgCl_2$, 0.2 mM DTT and increasing NaCl concentrations (0, 50, 100, 150 mM). Data sets were measured one time (no independent replicates).

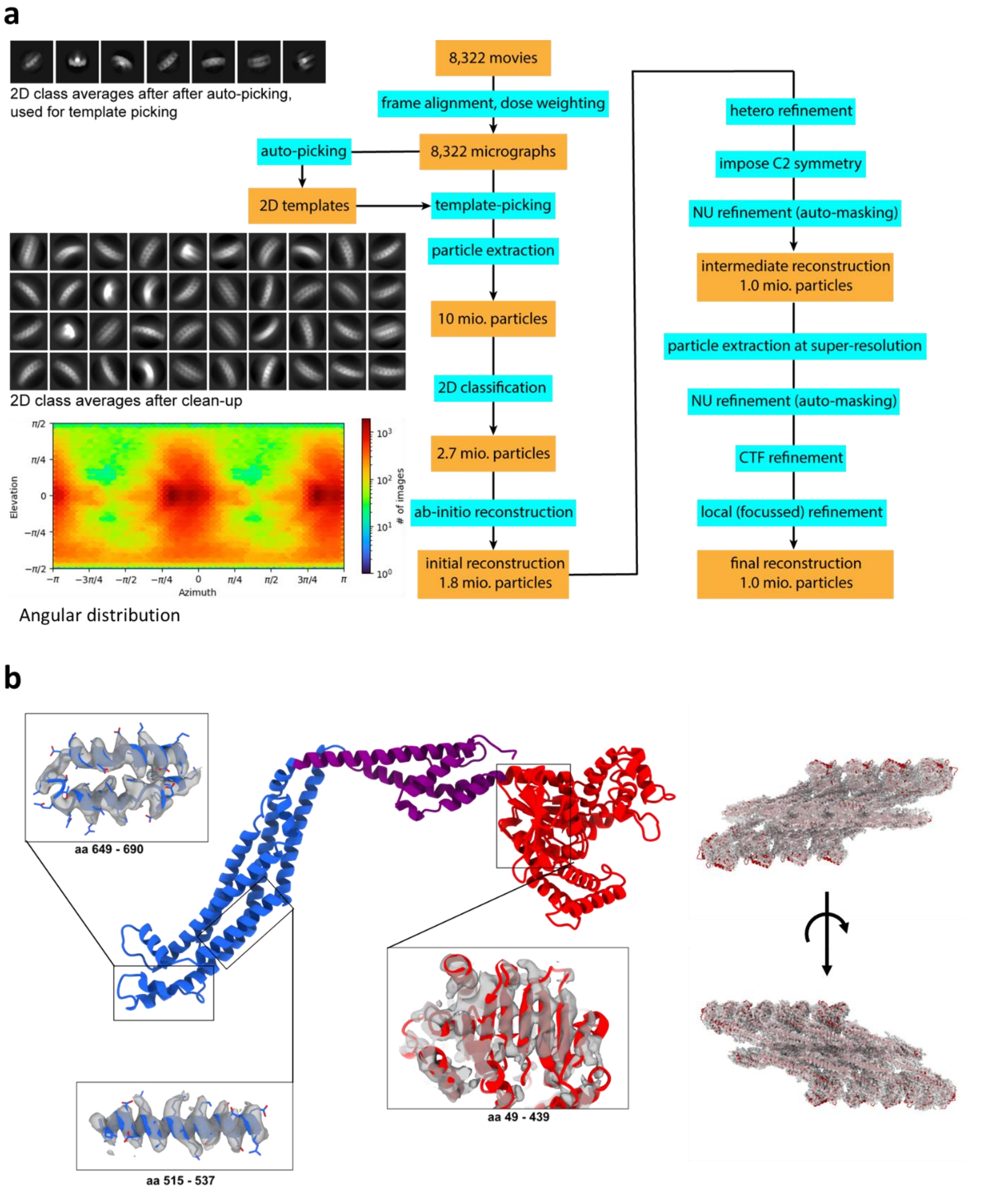

Supplementary Figure 5: Experimental details of the structure determination of *Syn*DLP oligomers.

(a) Flowchart of the applied data processing routines during *Syn*DLP structure determination using cryo-EM. (b) Model of the *Syn*DLP monomer, including insets showing the density fit of aa´s 649-690, 515-537, and 49-439 (left) and model fit to map from two viewing angles (right).

```
        10         20         30         40         50         60
MSKIAPQCQN LREQVNQLIE LLRQEPTLRS QQDTSIVETA LGKALSPRFE IVFAGAFSAG

        70         80         90        100        110        120
KSMLINALLE RELLYSAEGH ATGTECHIEY ANANEERVVL TFLSEAEIRQ QALILAKYLN

       130        140        150        160        170        180
VNVGDLNINQ PEAVKVVSQY CQKIIAEEGG ENKSERAKQA NALHLLLIGF EQNRERINTV

       190        200        210        220        230        240
QNSTYSMDQL NFSSLAEAAG YARRGANSAV LKRLDYFCNH SLLKDGNVLV DLPGIDAPVK

       250        260        270        280        290        300
EDAERAYRKI ESPDTSAVIC VLKPAAAGDM SAEETQLLER ISKNHGIRDR VFYVFNRIDD

       310        320        330        340        350        360
TWYNTQLRQR LEGLIQSQFR DNSRVYKTSG LLGFYGSQVK QTNSSTRFGL DSIFATTIKG

       370        380        390        400        410        420
FDGEEETPQF VSEFNNYCAN SGKLLSTAFR VSVNGYETSN ENYVRILSEW GIPLVDQLIH

       430        440        450        460        470        480
DSGIESFRSG IGLYLAEEKY PELFATLAND LQPLCIALRQ FYLENYRQLD SQPREIAAMK

       490        500        510        520        530        540
AQELTLLNQE MQNLGIEFKK YMSAQINDVV IGNDREFDQD FTKLKARMVA RLDELLKTFS

       550        560        570        580        590        600
VMNAYKRATE SHPRNSTAPF IAVLVEALYY LANELEDAFI EAIHELVKNF FQRLGDRLRK

       610        620        630        640        650        660
VDCYHQVYRL VGNDGGIEQL LRRAEEDITK ALVNEARTEC DRYVRESPRF YDEGTFSIYQ

       670        680        690        700        710        720
FRQTLQQTSQ GYDAQAIVEA EPAIKELLKL DFEPKVFNTV RKNFRQTVNN TLKTHLLPMA

       730        740        750        760        770        780
EEQAQIILEQ YDVARKYREQ TLEQDAEEKI ARNSRLQSEI KQKIDLYQTS IVSINECLKA

       790        800        810
MQIFEQLPVI TESDITKQAE IVADADFVEI VE
```

Supplementary Figure 6: Annotated amino acid sequence of *Syn*DLP.

*Syn*DLP domains are highlighted derived from the structure (Fig. 1): BSE1-3 in violet, GD in red, stalk in blue. The four substituted residues in the *Syn*DLP$_{HPRN\text{-}AAAA}$ mutant are typed orange and underlined. The two cysteines forming an intramolecular disulfide bridge are marked in yellow. The crucial active site residue K61 is highlighted in cyan. The two putative MIDs investigated in this study are marked in green or magenta, respectively.

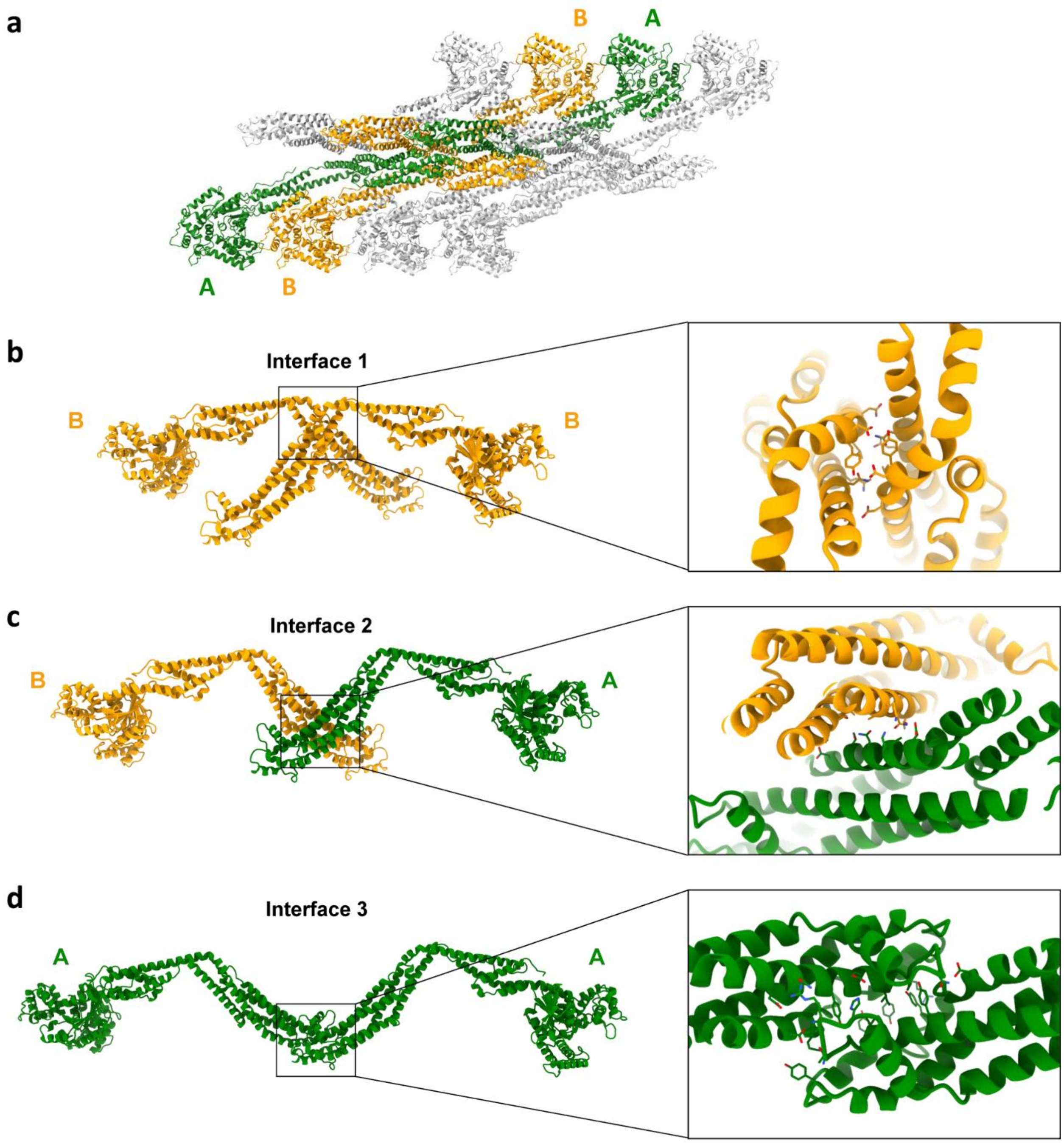

Supplementary Figure 7: Oligomerization interfaces in *Syn*DLP.

(a) *Syn*DLP structure in ribbon representation illustrating the position of the monomers shown in (b) – (d) within the oligomer in the same colorization. Isolated dimers of the *Syn*DLP oligomer are shown in ribbon representation and highlight oligomerization (b) interface 1, (c) interface 2 and (d) interface 3 including molecular details with intermolecular contact residues shown as sticks and colored by element in the zoomed sections. Monomers are colored in green and orange, respectively.

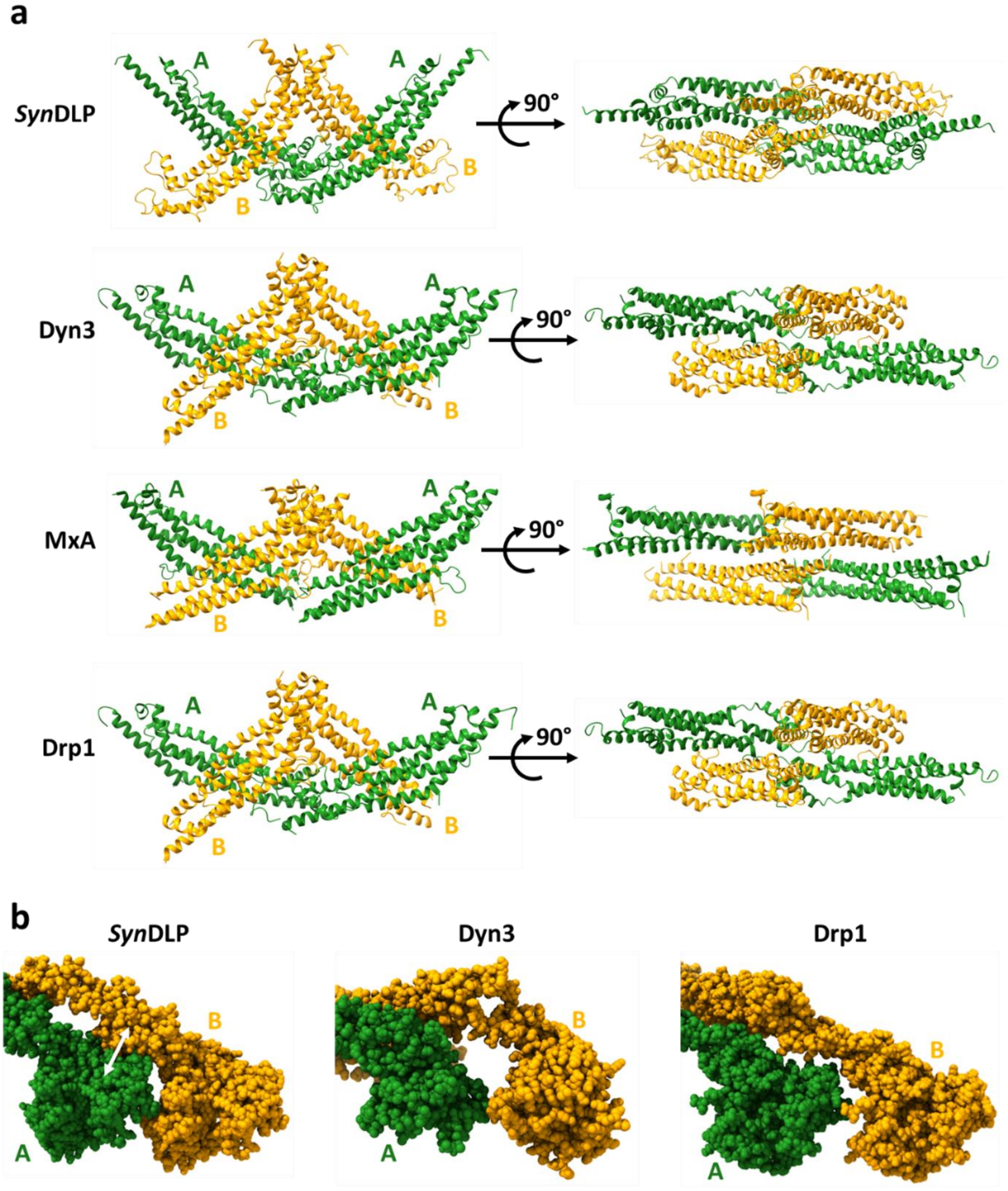

Supplementary Figure 8: Comparison of oligomeric interfaces in different DLPs.

(a) Tetramers of *Syn*DLP (this study), Dyn3 (PDB: 5A3), MxA (PDB: 3LJB) and Drp1 (PDB: 5WP9) in ribbon representation showing common oligomerization interfaces in the stalk domain. Monomers are marked in green and orange, respectively. The GD and the BSE are omitted for clarity. (b) Longitudinal GD-GD contacts and the intermolecular GD-BSE interface of *Syn*DLP is compared to Dyn3 and Drp1. GD and BSE of two adjacent monomers within the oligomers are shown. The two monomers are colored green and orange, respectively. The enlarged GD-BSE interface in *Syn*DLP is highlighted by a white arrow.

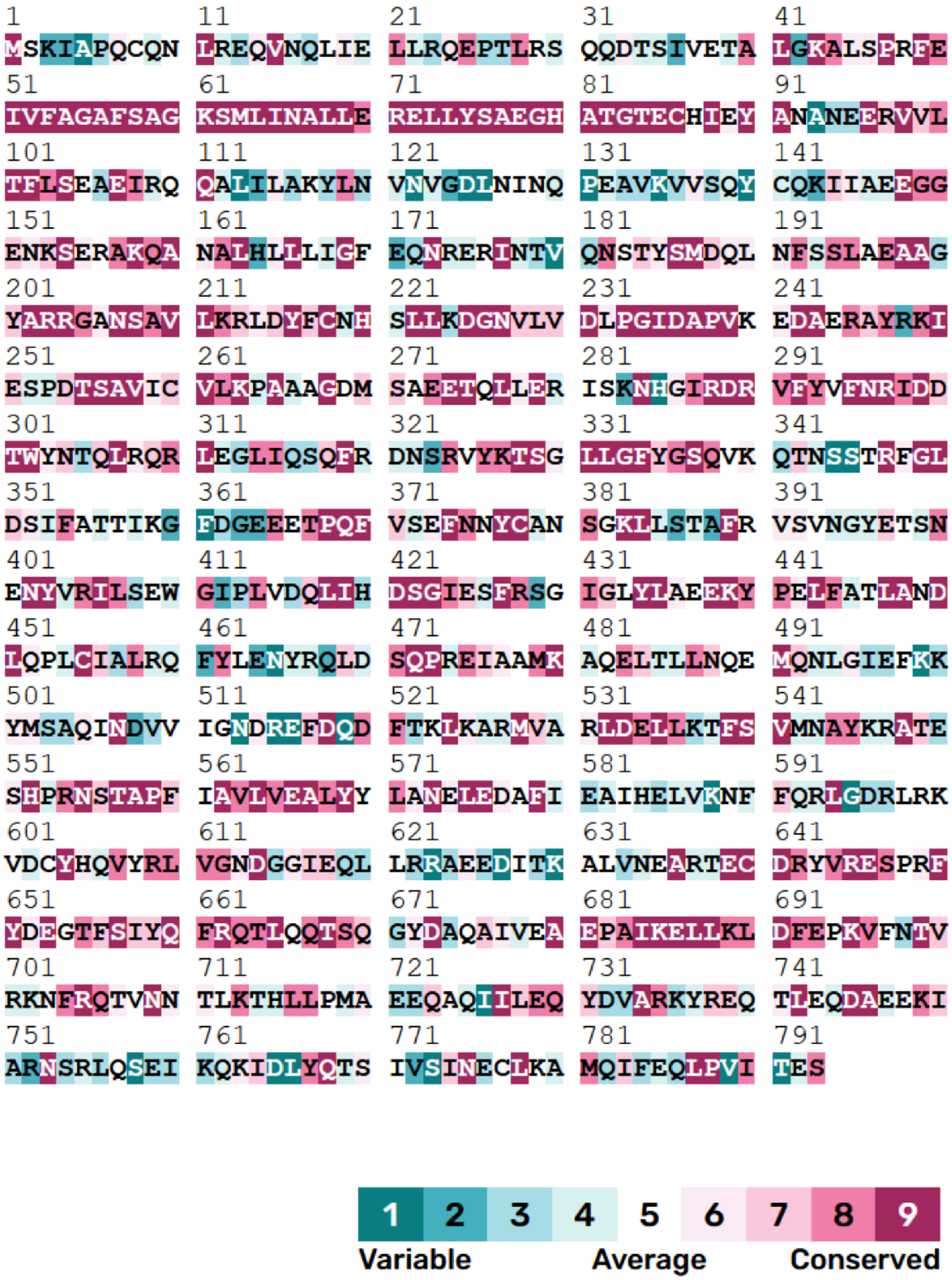

Supplementary Figure 9: Conservation of single residues in *Syn*DLP.

The primary sequence of *Syn*DLP is shown. The conservation of single *Syn*DLP residues was evaluated based on a sequence alignment with 150 related DLP sequences selected by the online tool ConSurf[3–7]. Amino acids are colored from dark cyan (variable) to red-violet (conserved).

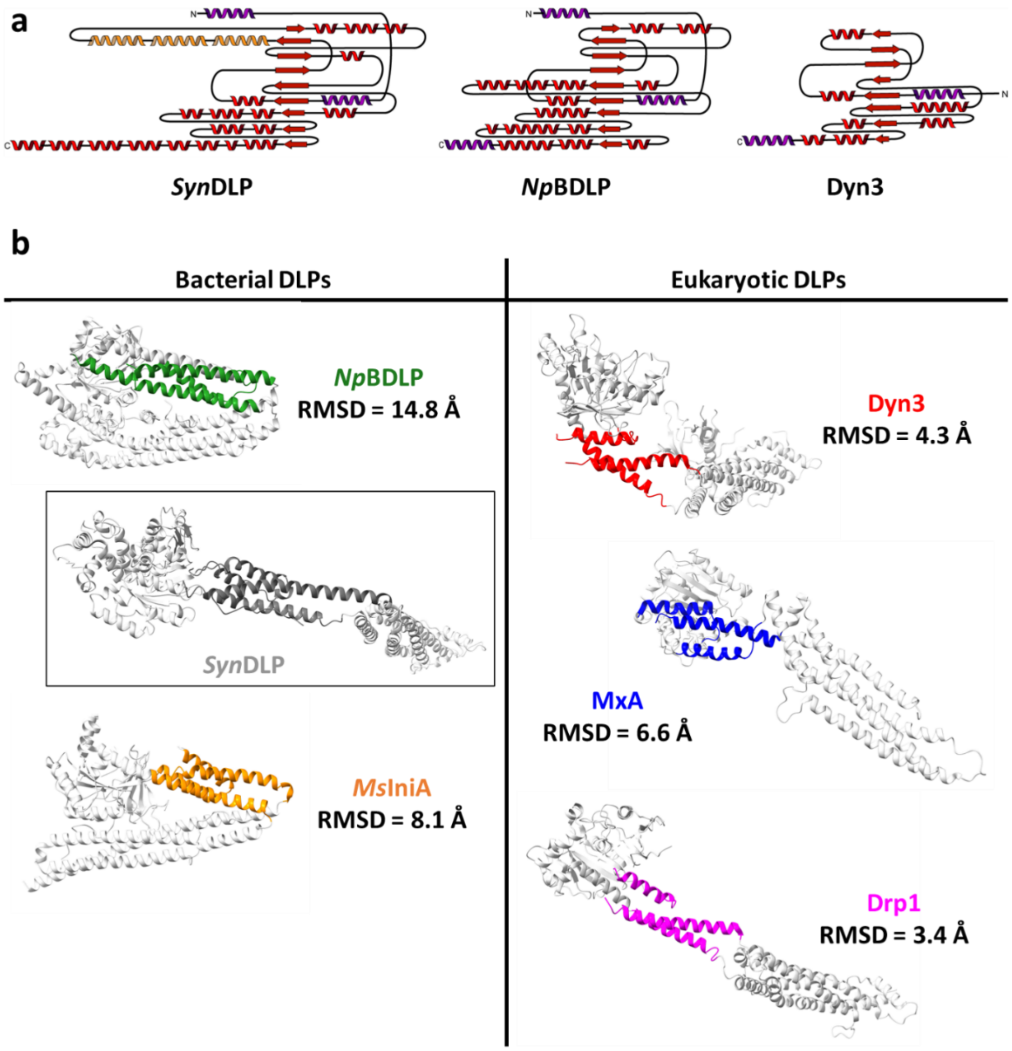

c

		GD		BSE		Stalk	
		# aligned aa	RMSD [Å]	# aligned aa	RMSD [Å]	# aligned aa	RMSD [Å]
Bacterial DLPs	*Np*BDLP	219	10.8	63	14.8	157	29.6
	*Ms*IniA	222	11.8	61	8.1	132	14.3
Eukaryotic DLPs	Dyn3	252	16.1	29	4.3	105	10.2
	MxA	221	18.7	42	6.6	152	8.5
	Drp1	249	15.8	33	3.4	106	30.8

Supplementary Figure 10: Structural comparison of DLP domains.

(a) Topology plots of the GDs derived from the *Syn*DLP structure, *Np*BDLP (PDB: 2J69) and Dyn3 (PDB: 5A3F). α-helices and β-sheets of the GD are colored in red and α-helices of the BSE domain in purple. The additional α-helices of the *Syn*DLP GD are colored in orange. (b) In the monomer model of *Syn*DLP, the structure of the BSE (gray) was aligned with the BSE of the BDLPs *Np*BDLP (PDB: 2J69, green) and *Ms*IniA (PDB: 6J73, orange) and with the eukaryotic representatives Dyn3 (PDB: 5A3F, red), MxA (PDB: 3SZR, blue) and Drp1 (PDB: 5WP9, magenta). RMSD values are given. The regions used for the alignments are highlighted by color and the remaining protein regions are colored in light gray. (c) A table that summarizes the structural alignments performed in this study. An indicated number of amino acids from the domains of the *Syn*DLP monomer structure was aligned to the respective domains of the corresponding bacterial and eukaryotic DLPs leading to the Cα-RMSD values.

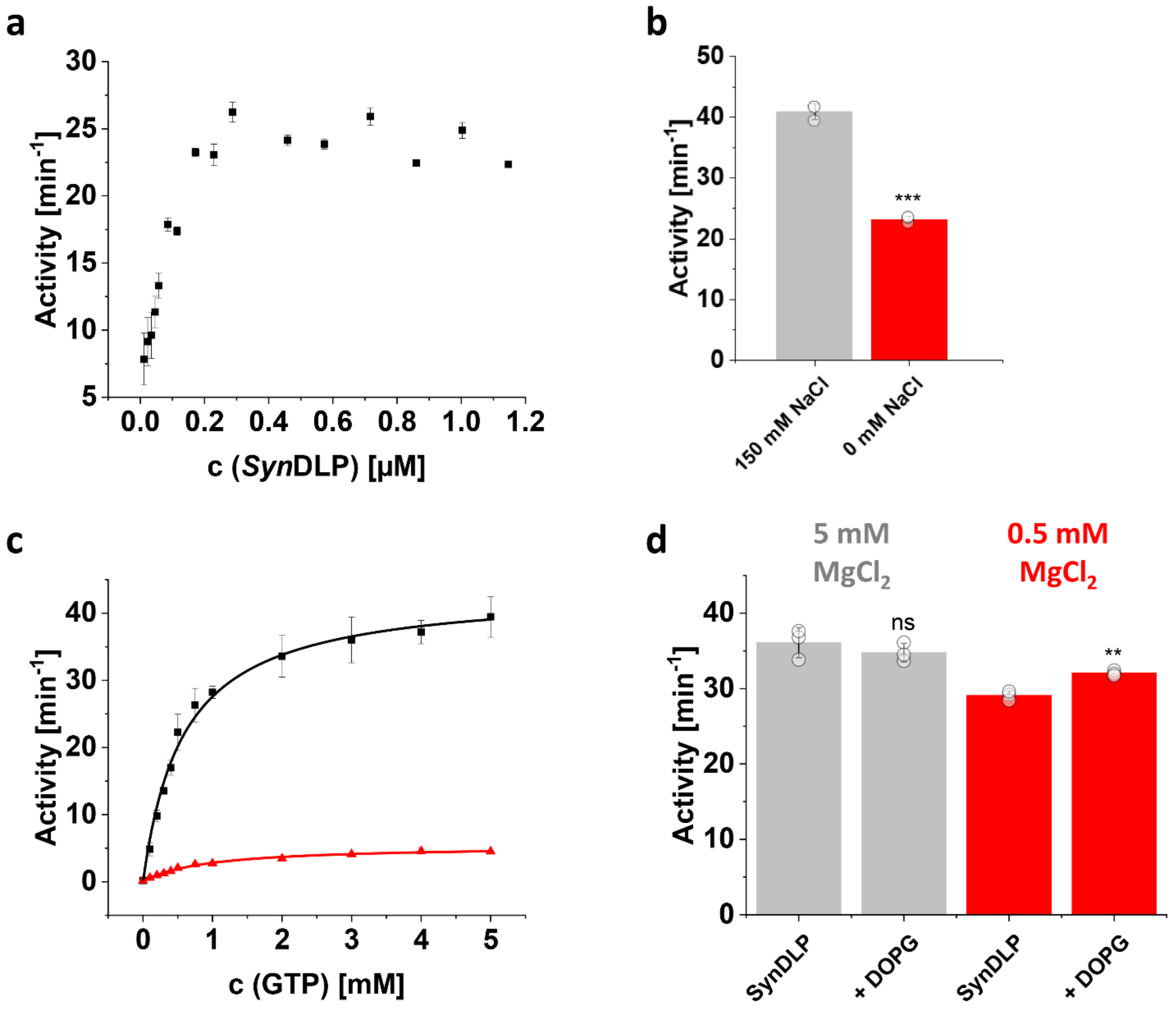

Supplementary Figure 11: The *Syn*DLPs GTPase activity.

(a) The GTPase activity of increasing *Syn*DLP concentrations (black) was measured in a continuous, regenerative, coupled assay at a GTP concentration of 1 mM. Mean of independent measurements (n=3) and S.D. are shown. (b) GTPase activity of *Syn*DLP wt was measured at a GTP concentration of 5 mM under standard conditions (presence of 150 mM NaCl, gray) and in the absence of NaCl (red). Mean of three measurements and error bars (S.D.) are shown. Single measurements are shown as circles. ns = not significant ($P > 0.05$), * $P < 0.05$, ** $P < 0.01$, *** $P < 0.001$ based on a two-sided unpaired Student's t-test. $P = 0.000025$. (c) The GTPase activities of 0.5 µM *Syn*DLP wt (black) and *Syn*DLP$_{K61A}$ (red) were measured. Mean of three measurements and error bars (S.D.) are shown. The kinetic parameters of the *Syn*DLP$_{K61A}$ mutant were determined as $k_{cat} = 5.4 \pm 0.2$ min^{-1} and $K_m = 0.91 \pm 0.07$ min^{-1} (for *Syn*DLP wt see Table 3) (d) GTPase activity of *Syn*DLP wt was determined at 5 mM GTP in the absence or presence of 20 µM DOPG LUVs (extruded to 100 nm) either with 5 mM (gray) or 0.5 mM $MgCl_2$ (red). LUVs were added with the assay components. Mean of three measurements and error bars (S.D.) are shown. Single measurements are displayed as circles. ns = not significant ($P > 0.05$), * $P < 0.05$, ** $P < 0.01$, *** $P < 0.001$ based on a two-sided unpaired Student's t-test. The activity in the absence of LUVs is compared to the activity of *Syn*DLP plus DOPG for the respective $MgCl_2$ concentrations (5 mM $MgCl_2$: $P = 0.38$; 0.5 mM $MgCl_2$: $P = 0.0018$).

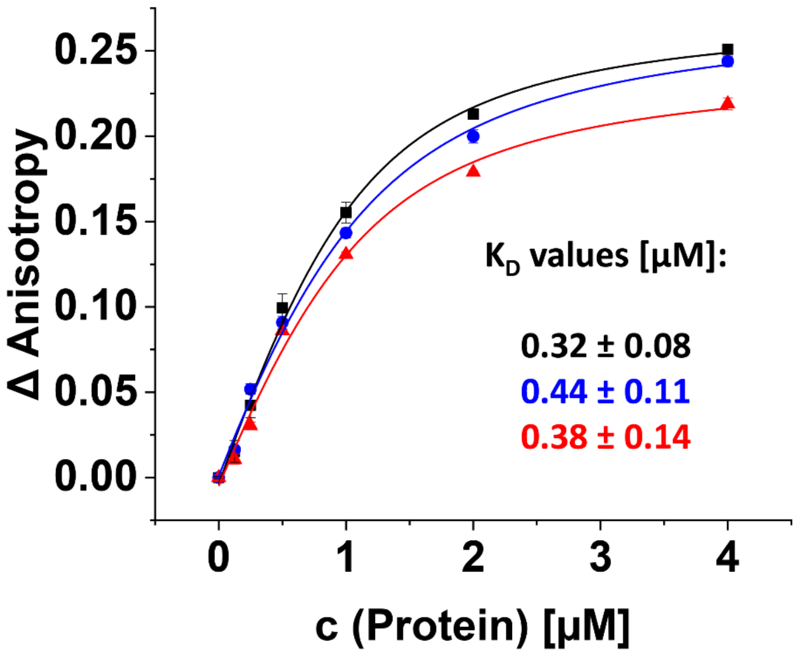

Supplementary Figure 12: GTP binding affinities of *Syn*DLP variants.

GTP binding of *Syn*DLP variants was measured using Mant-GTP (Jena Bioscience, Jena, Germany). Therefore, increasing concentrations of *Syn*DLP wt (black), *Syn*DLP$_{C777A}$ (blue) and *Syn*DLP$_{HPRN\text{-}AAAA}$ (red) were incubated with 1 µM Mant-GTP in reaction buffer at 20°C for 30 min. The change in the fluorescence anisotropy was measured ten times (λ_{ex} = 355 nm, λ_{em} = 448 nm, slit widths corresponding to 7 nm and 7 nm, T = 20°C) using a Fluoromax-4 spectrometer (Horiba Scientific, Kyoto, Japan) and the results averaged. The data points show mean of independent experiments (n=3) and standard deviation and were fitted using a quadratic binding equation assuming a one-site specific binding model (Supplementary Equation (1)):

$$r\,(x) = r_0 + r_{max} * \frac{(F+x+K_D)-\sqrt{(F+x+K_D)^2-4*x*F}}{2*F} \qquad (1)$$

Here, r corresponds to the anisotropy, r_0 to the anisotropy without protein, r_{max} is the maximum anisotropy, F is the fluorophore concentration and x the protein concentration. K_D refers to the dissociation constant. The fits are shown as lines and the K_D values from each fit are shown with S.D. colored corresponding to the data points of the protein variant.

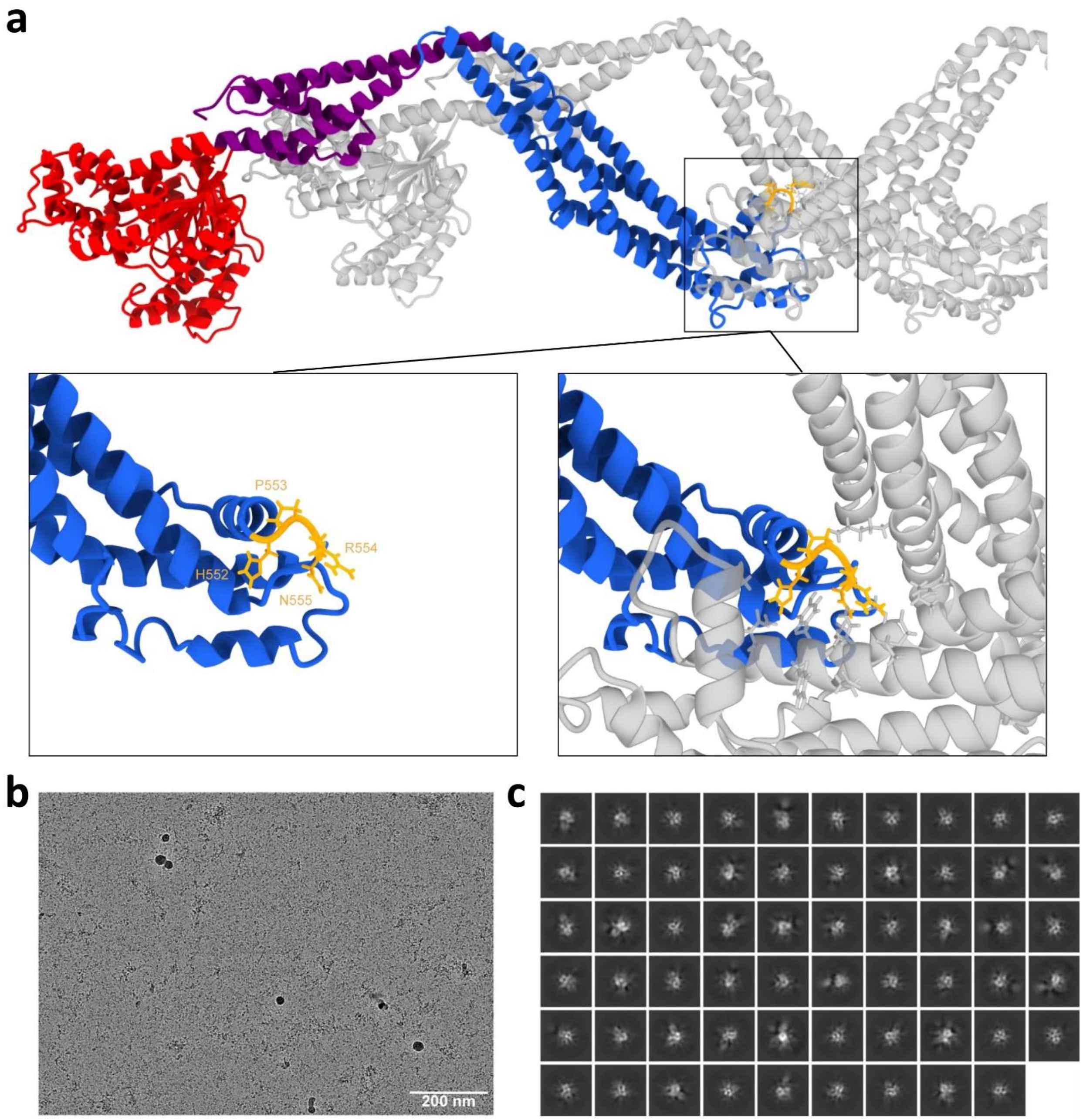

Supplementary Figure 13: Mutation of oligomerization interface 3 residues.

(a) An isolated tetramer of the *Syn*DLP oligomer is shown as ribbon representation in grey. The domains are colored in one monomer (GD in red, BSE in purple, stalk in blue). The zoomed sections show four residues (^{552}HPRN^{555}) colored in orange and as sticks that lie on a loop in the oligomerization interface 3 either in one monomer (left) or together with contacting monomers (right). ^{552}HPRN^{555} were substituted to AAAA to impair the assembly of *Syn*DLP oligomers. (b) Cryo-EM micrograph of *Syn*DLP$_{\text{HPRN-AAAA}}$. The entire protein sample before analytical gel filtration (see Fig. 4c) was analyzed (no single fractions). Data set was measured one time (no independent replicates). (c) 2D class averages of *Syn*DLP$_{\text{HPRN-AAAA}}$ particles (250,000 particles with 272 Å box dimension).

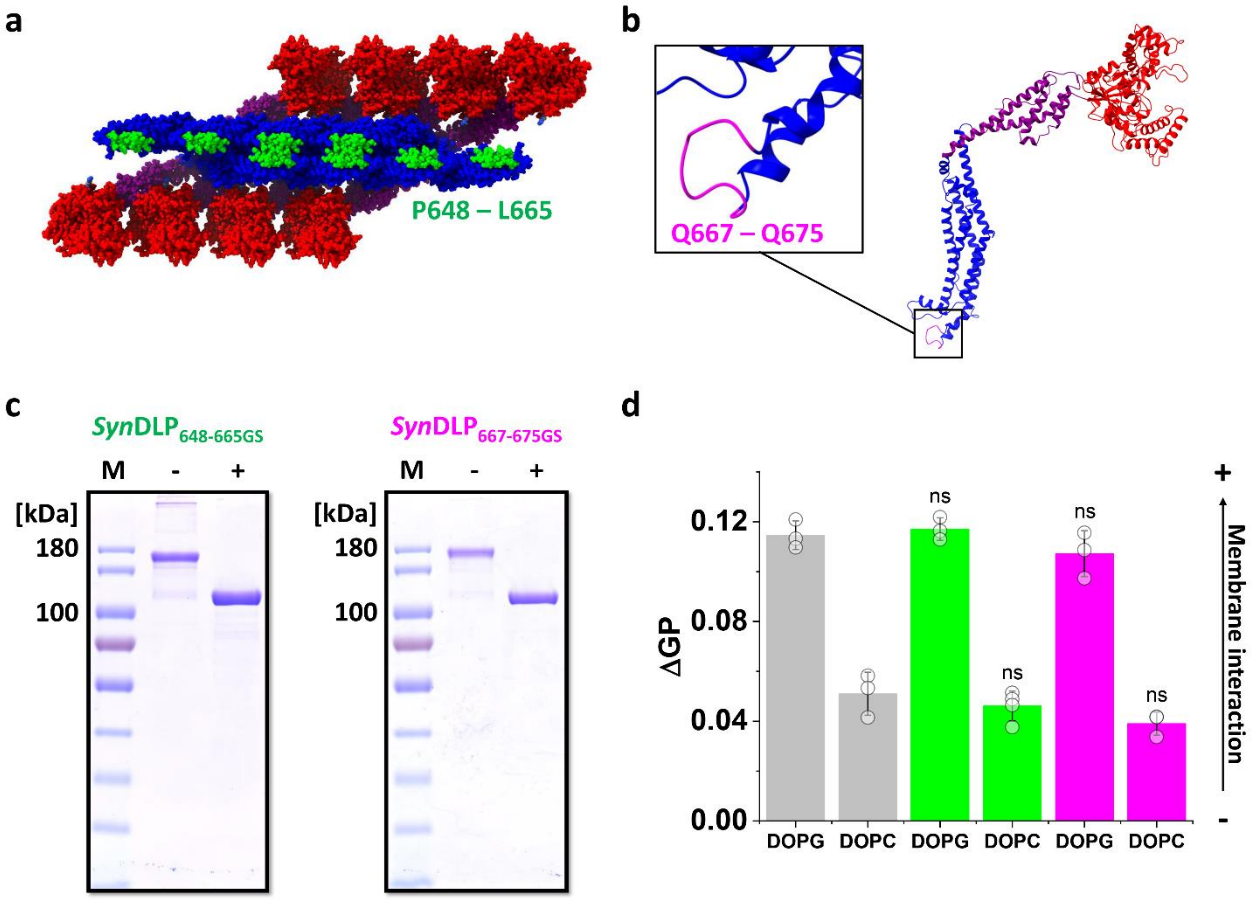

Supplementary Figure 14: Putative membrane interaction domains in *Syn*DLP.

Putative membrane interaction sites in the *Syn*DLP structure were predicted according to the position of amino acid residues at the tip of the stalk domain. (a) Based on the quaternary structure of *Syn*DLP oligomers, a domain consisting of 18 residues (P648 – L665) was identified being a putative MID. A *Syn*DLP oligomer is shown with the GD, BSE, stalk and the putative MID colored in red, blue, purple and green, respectively. (b) Another putative MID was determined as a 9 amino acid loop (Q667 – Q675) based on the tertiary structure of a *Syn*DLP monomer. A *Syn*DLP monomer is shown in ribbon representation, coloring as in (a), except that the putative MID is highlighted in magenta. (c) To investigate the influence of both assumed MIDs on the membrane binding properties of *Syn*DLP, the respective amino acids were substituted by multiple repetitions of glycine and serine (GS-linker), leading to the mutant proteins *Syn*DLP$_{648\text{-}665GS}$ and *Syn*DLP$_{667\text{-}675GS}$. The two SDS-PAGE gels showed that the purity of the recombinantly expressed proteins (calculated molecular masses in each case 93 Da), which were purified as described for *Syn*DLP wt, is ≥ 95% (lane +: presence of 100 mM DTT). Under non-reducing conditions (0.1 mM DTT, lane -) both mutant proteins migrated at higher molecular masses, indicating correct formation of the intramolecular disulfide bridge established in the BSE domain of *Syn*DLP. M = marker. Representative gels of two independent experiments showing the same results. (d) Membrane binding of the assumed membrane interaction-defective mutants was analyzed and compared to *Syn*DLP wt via fluorescence spectroscopy using Laurdan as a fluorescent probe. LUVs contained either 50% DOPC and 50% DOPG (w/w), which was shown to promote strong interaction with *Syn*DLP wt (Fig. 5a), or 100% DOPC, each time mixed with Laurdan at a 1:500 molar ratio. Fluorescence emission spectra of Laurdan were measured after 30 min incubation of the proteins and LUVs. ΔGP values were calculated from the spectra. The mean of three measurements and error bars (S.D.) are shown. Single measurements are shown as circles. ns = not significant ($P > 0.05$), * $P < 0.05$, ** $P < 0.01$, *** $P < 0.001$ based on a two-sided unpaired Student's t-test. The ΔGP value of either *Syn*DLP$_{648\text{-}665GS}$ or *Syn*DLP$_{667\text{-}675GS}$ is compared to *Syn*DLP wt plus DOPG or DOPC, respectively. The arrow indicates increasing membrane interaction. Determined ΔGP values of *Syn*DLP wt, *Syn*DLP$_{648\text{-}665GS}$ and *Syn*DLP$_{667\text{-}675GS}$ are shown as bars colored in grey, green or magenta, respectively.

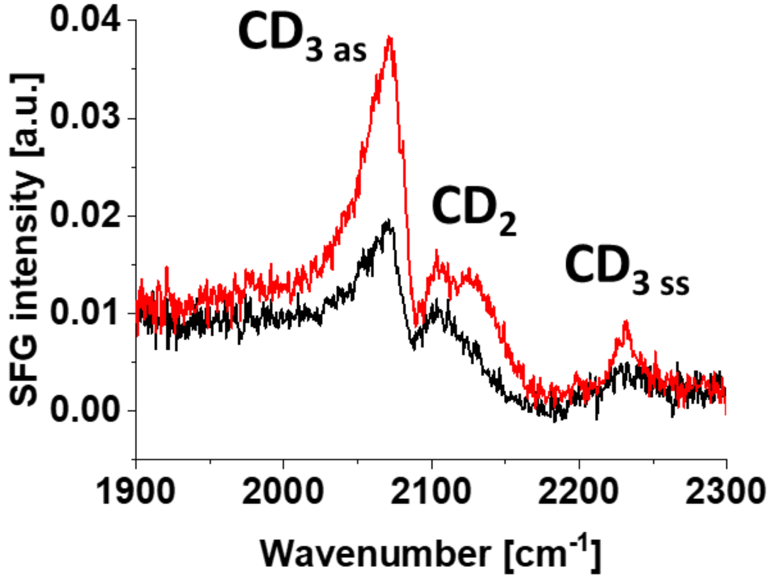

Supplementary Figure 15: SFG spectra of a deuterated DMPG monolayer.

SFG spectra of a deuterated DMPG monolayer were recorded in the C-D stretching region before (black) and after (red) *Syn*DLP binding. The surface pressure was set to 15 mN/m for the pure monolayer. Modes related to the asymmetric (as) and symmetric (ss) stretching mode of the terminal methyl group as well as the chain methylenes are visible. The increase of the signal after *Syn*DLP binding shows that the protein partly intercalates with the lipid acyl chains, which increases the molecular alignment.

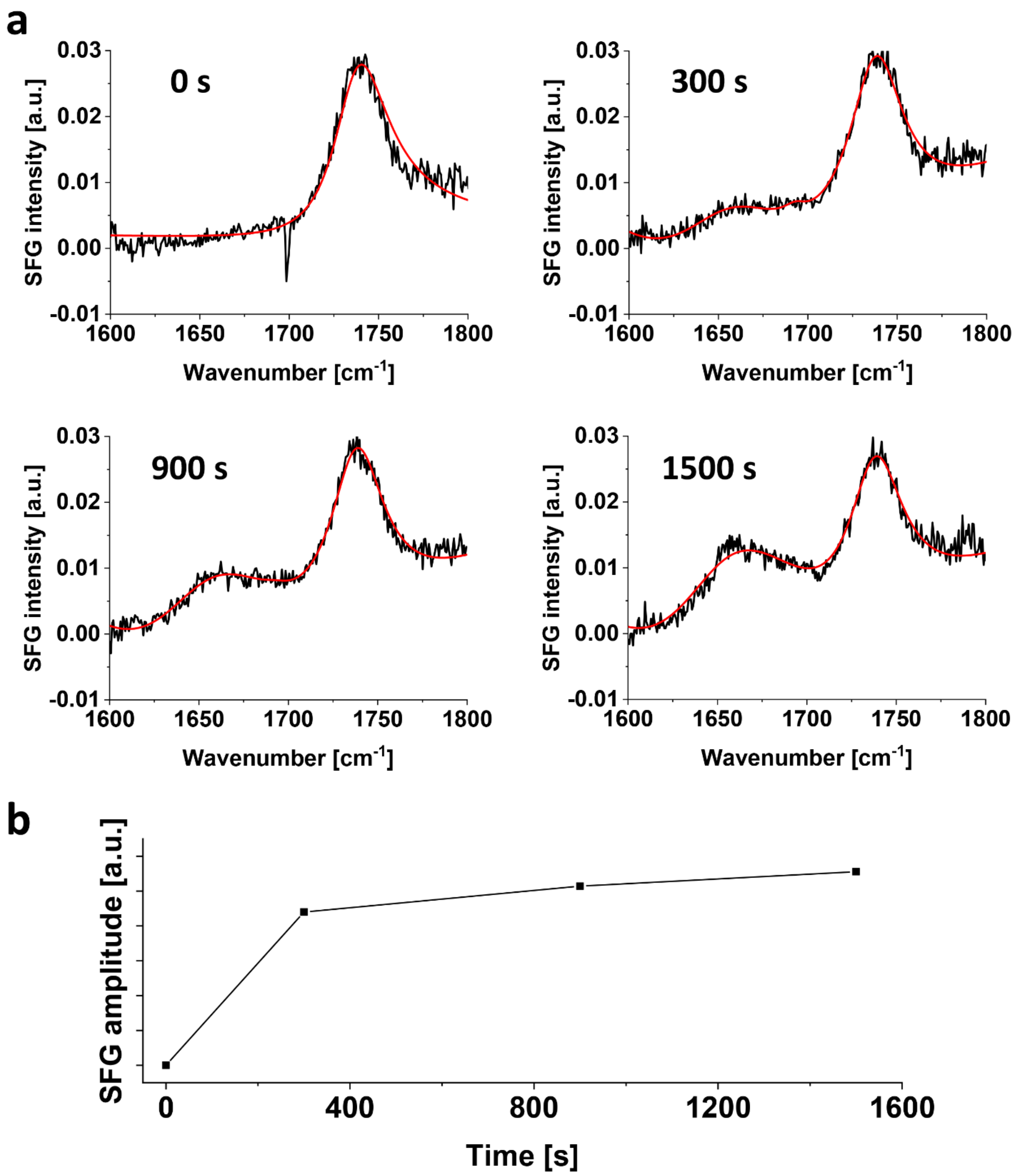

Supplementary Figure 16: Time-dependent SFG spectra of *Syn*DLP membrane binding.

(a) SFG amide I spectra (black) of *Syn*DLP binding to a DMPG monolayer recorded at different times after the protein injection. Fits are shown as red lines. While the lipid C=O resonance remains almost constant, the protein amide I mode is growing over time. (b) SFG amplitude (black) of the helical component of the SFG peak fit plotted as a function of the time after injection.

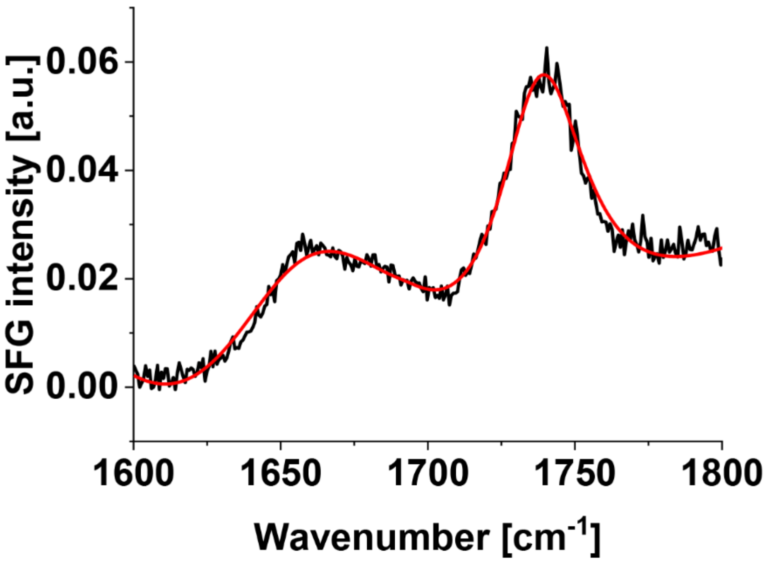

Supplementary Figure 17: SFG spectrum of membrane-bound *Syn*DLP with GTP.

SFG amide I spectrum of *Syn*DLP bound to a DMPG monolayer in the presence of GTP in solution (black). A fit is displayed as a red line.

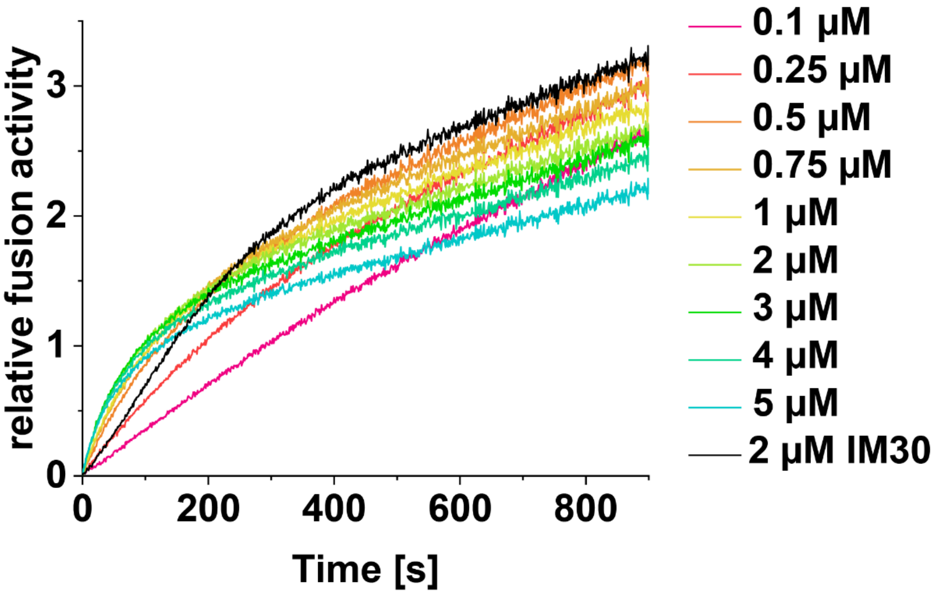

Supplementary Figure 18: Membrane fusion curves of *Syn*DLP and IM30.

Curves show the entire measurement over 900 s with increasing *Syn*DLP concentrations as well as the positive control (2 µM IM30, black) as described in Fig. 7b. The curves represent the average of three measurements.

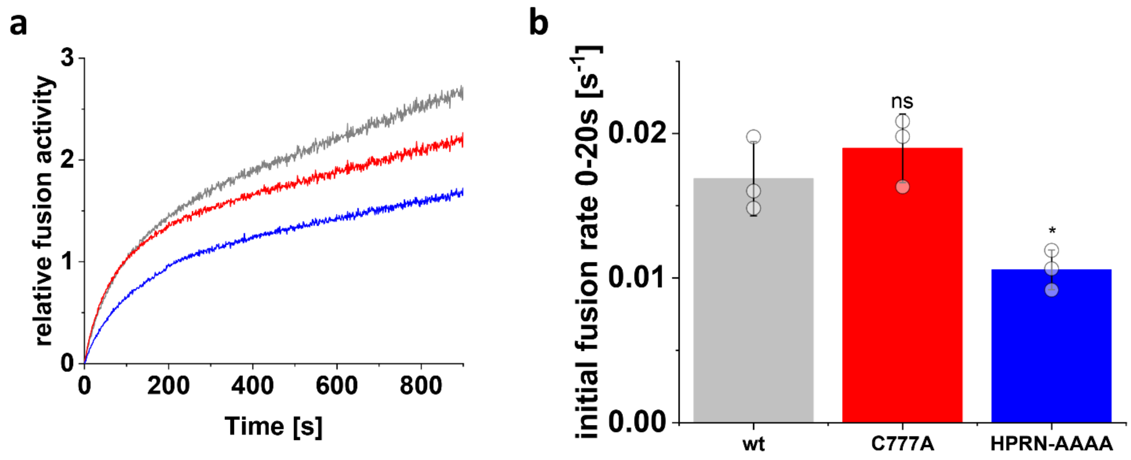

Supplementary Figure 19: Membrane fusion activity of *Syn*DLP variants.

Fusion of MGDG/DOPG (60%/40%, w/w) LUVs was measured in a FRET-based fusion assay. (a) Comparison of the fusion curves using 2 µM *Syn*DLP wt (gray), *Syn*DLP$_{C777A}$ (red) or *Syn*DLP$_{HPRN-AAAA}$, respectively (blue). Calculation of the relative fusion activities is described in the methods section. The curves represent mean of independent measurements (n=3). (b) From the fusion curves in (a) initial fusion rates were defined as the slope of a linear regression of the first 20 s. Mean of three measurements and error bars (S.D.) are shown and compared to the wt. Single measurements are displayed as circles. ns = not significant ($P > 0.05$), * $P < 0.05$, ** $P < 0.01$, *** $P < 0.001$ based on a two-sided unpaired Student's t-test. The initial fusion rate of the wt is compared to *Syn*DLP$_{C777A}$ ($P = 0.36$) or *Syn*DLP$_{HPRN-AAAA}$ ($P = 0.02$), respectively. Colors as in (a).

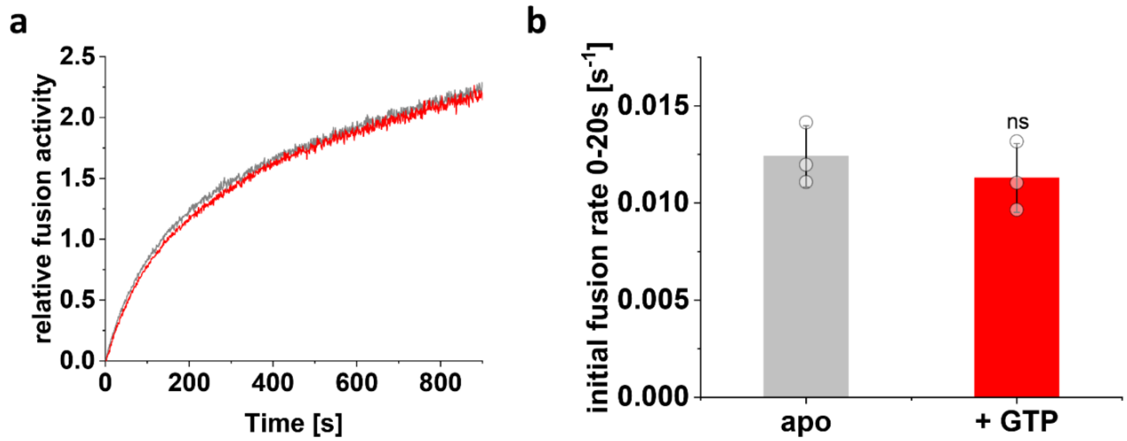

Supplementary Figure 20: Influence of GTP on the membrane fusion activity.

In a FRET-based assay, fusion of MGDG/DOPG (60%/40%, w/w) LUVs was measured. (a) Fusion curves determined with 2 µM *Syn*DLP in the absence (gray) or presence of 1 mM GTP (red). The measurements with GTP were performed with 7 mM $MgCl_2$ (instead of 5 mM $MgCl_2$) to compensate for nucleotide addition. Moreover, for the calculation of the relative fusion activities (described in method section) an additional negative control including 1 mM GTP and 7 mM $MgCl_2$ was measured for the sample with GTP. Mean of three independent measurements is shown. (b) Initial fusion rates were calculated via taking the slope from a linear regression of the first 20 s of the fusion curves shown in (a). Mean of independent experiments (n=3) and S.D. are displayed. Single measurements are shown as circles. ns = not significant ($P > 0.05$), * $P < 0.05$, ** $P < 0.01$, *** $P < 0.001$ based on a two-sided unpaired Student's t-test. Coloring is the same as in (a).

SynDLP	4	IAPQCQNLR	…	773	SINECLKAM
Cyan8802_4244	4	LVPQCQNLS	…	772	GINGCLQAM
Cylst_3537	4	LAPQCQQLV	…	771	EINSCLQAM
WP_006530685	4	TPADCQHLP	…	766	GVNNCLKAM
Mic7113_0538	4	ILPQCQNLQ	…	782	GINSCLQGM
Nos7524_0596	18	LPFQCHNLK	…	785	HINGCLQAM
HLUCCO16_19355	4	TPPQCQNLD	…	775	GINGCLEEM
Riv7116_3732	4	LLPQCQNLK	…	769	SINGCLSAM
Syn6312_2093	4	LTHLCPDLQ	…	772	SINSCLELM
DA73_0213440	4	LPLQCKNLQ	…	771	SINECLEAV
Consensus		xxxQCxxLx			xINxCLxxM

Sequence logo

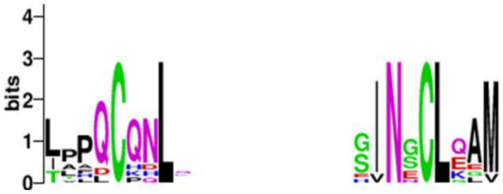

Supplementary Figure 21: Disulfide forming cysteine residues are conserved within cyanobacterial KGK clade DLPs.

Sequence alignment of predicted cyanobacterial dynamin-like GTPases belonging to the KGK clade. The section shows the sequence context of *Syn*DLP C8 and C777 residues both colored in green together with corresponding sequence regions of other cyanobacterial KGK clade DLPs. Cyan8802_4244 from *Cyanothece* sp. PCC 8802, Cylst_3537 from *Cylindrospermum stagnale* PCC 7417, WP_006530685 from *Gloeocapsa* sp. PCC 73106, Mic7113_0538 from *Microcoleus* sp. PCC 7113, Nos7524_0596 from *Nostoc* sp. PCC 7524, HLUCCO16_19355 from *Phormidium* sp. OSCR, Riv7116_3732 from *Rivularia* sp. PCC 7116, Syn6312_2093 from *Synechococcus* sp. PCC 6312, DA73_0213440 from *Tolypothrix bouteillei* VB521301.

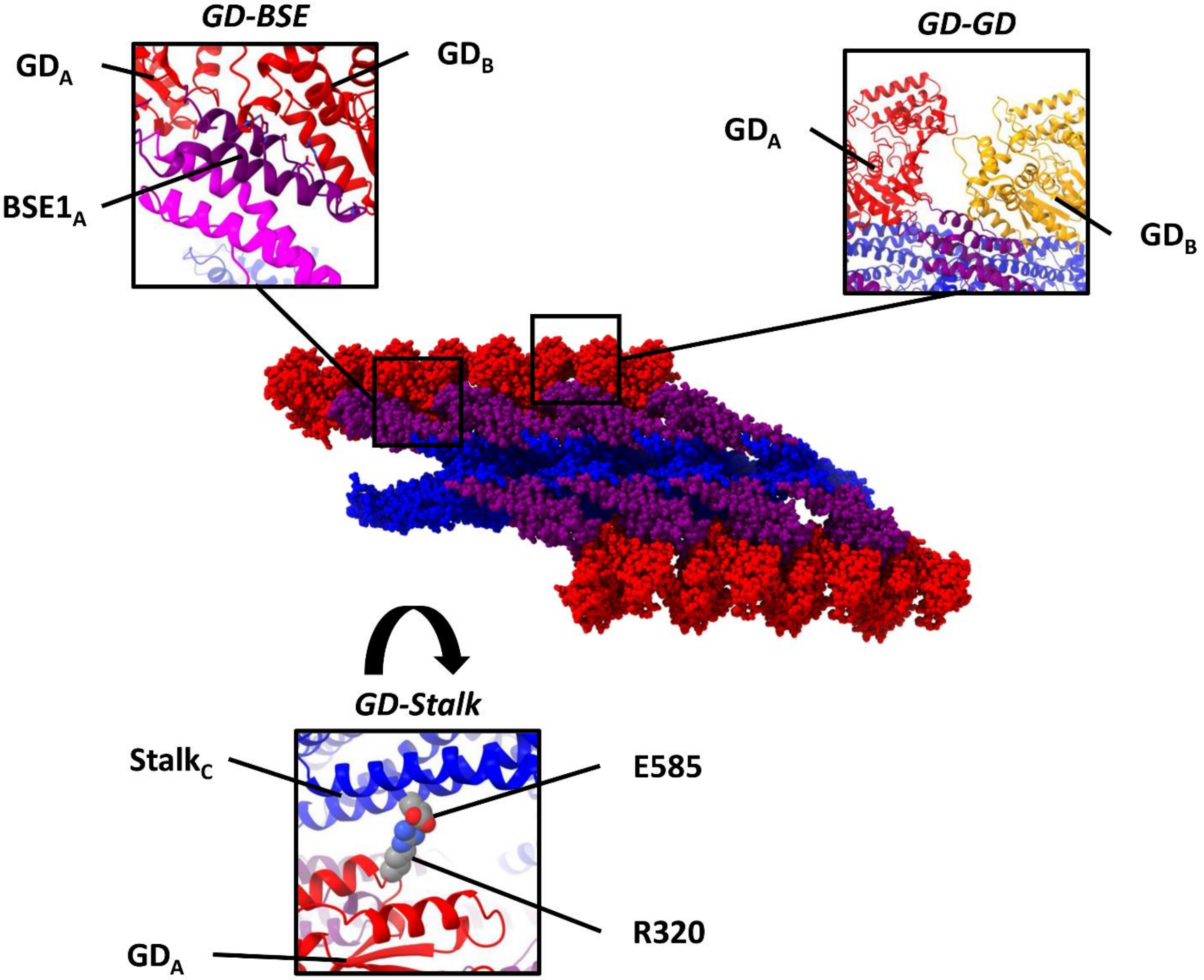

Supplementary Figure 22: Intermolecular domain contacts in the *Syn*DLP oligomer.

Structure of a *Syn*DLP oligomer is shown in the center. GD, BSE and stalk are colored red, violet and blue, respectively. The three sections zoom areas of contacts of one GD with another polypeptide chain, either between the GD and the BSE domain (GD-BSE; BSE1 colored in purple, BSE2 and BSE3 in magenta; contact residues from the BSE1 domain shown as sticks), between two GDs (GD-GD; GD_A and GD_B colored in red and orange, respectively) or between the GD and the stalk domain (GD-stalk; residues mediating GD-stalk contacts (R320 and E585) are shown as spheres and colored by element).

Supplementary References

1. Rippka, R., Deruelles, J., Waterbury, J. B., Herdman, M. & Stanier, R. Y. Generic assignments, strain histories and properties of pure cultures of cyanobacteria. *J. Gen. Microbiol.* **111**, 1–61 (1979).

2. Schindelin, J. *et al.* Fiji: an open-source platform for biological-image analysis. *Nat. Methods* **9**, 676–682 (2012).

3. Celniker, G. *et al.* ConSurf: using evolutionary data to raise testable hypotheses about protein function. *Isr. J. Chem.* **53**, 199–206 (2013).

4. Glaser, F. *et al.* ConSurf: identification of functional regions in proteins by surface-mapping of phylogenetic information. *Bioinformatics* **19**, 163–164 (2003).

5. Ashkenazy, H. *et al.* ConSurf 2016: an improved methodology to estimate and visualize evolutionary conservation in macromolecules. *Nucleic Acids Res.* **44**, W344–W350 (2016).

6. Landau, M. *et al.* ConSurf 2005: the projection of evolutionary conservation scores of residues on protein structures. *Nucleic Acids Res.* **33**, W299–W302 (2005).

7. Ashkenazy, H., Erez, E., Martz, E., Pupko, T. & Ben-Tal, N. ConSurf 2010: calculating evolutionary conservation in sequence and structure of proteins and nucleic acids. *Nucleic Acids Res.* **38**, W529–W533 (2010).
