# Supplementary material for: SynDLP is a dynamin-like protein of Synechocystis sp. PCC 6803 with eukaryotic features
Source: Nat Commun. 2023 Apr 14;14:2156. doi: 10.1038/s41467-023-37746-9 (PMC10104851; doi:10.1038/s41467-023-37746-9)
Supplement: Supplementary file 3 — Reporting Summary [file 41467_2023_37746_MOESM3_ESM.pdf]

nature portfolio

Corresponding author(s): Carsten Sachse, Dirk Schneider

Last updated by author(s): Mar 20, 2023

Reporting Summary

Nature Portfolio wishes to improve the reproducibility of the work that we publish. This form provides structure for consistency and transparency in reporting. For further information on Nature Portfolio policies, see our Editorial Policies and the Editorial Policy Checklist.

Statistics

For all statistical analyses, confirm that the following items are present in the figure legend, table legend, main text, or Methods section.

n/a	Confirmed	
☐	☒	The exact sample size (*n*) for each experimental group/condition, given as a discrete number and unit of measurement
☐	☒	A statement on whether measurements were taken from distinct samples or whether the same sample was measured repeatedly
☐	☒	The statistical test(s) used AND whether they are one- or two-sided<br>*Only common tests should be described solely by name; describe more complex techniques in the Methods section.*
☐	☒	A description of all covariates tested
☐	☒	A description of any assumptions or corrections, such as tests of normality and adjustment for multiple comparisons
☐	☒	A full description of the statistical parameters including central tendency (e.g. means) or other basic estimates (e.g. regression coefficient) AND variation (e.g. standard deviation) or associated estimates of uncertainty (e.g. confidence intervals)
☐	☒	For null hypothesis testing, the test statistic (e.g. F, t, r) with confidence intervals, effect sizes, degrees of freedom and P value noted<br>*Give P values as exact values whenever suitable.*
☐	☒	For Bayesian analysis, information on the choice of priors and Markov chain Monte Carlo settings
☐	☒	For hierarchical and complex designs, identification of the appropriate level for tests and full reporting of outcomes
☒	☐	Estimates of effect sizes (e.g. Cohen's *d*, Pearson's *r*), indicating how they were calculated

Our web collection on statistics for biologists contains articles on many of the points above.

Software and code

Policy information about availability of computer code

Data collection	Omega (Version 1.3), UNICORN (Version 5.10), JASCO Spectra Manager (Version 2.9.0.7), JASCO Spectra Manager (Version 2.15.01), FluorEssence (Version 3.8), SFGTools (can be dowloaded at https://github.com/james-d-pickering/SFGTools), ZS Xplorer (Version 2.3.1.4), Digital Micrograph (Version 3.32.2403.0), EPU (Version 2.11.0.2368REL), TIA (Version 5.0.0.2896), FluCam viewer (Version 6.15.3.22415)
Data analysis	Origin (Version 9.60), MS Excel (Version 2208), MARS Data Analysis Software (Version 2.10 R3), JASCO Spectra Analysis (version 2.15.01), Matlab (Version 2022a), cryoSPARC (Version 3.2), cryoSPARC live (Version 3.2), ISOLDE (Version 1.2), Coot (Version 0.95), Phenix (Version 1.18), ChimeraX (Version 1.2.5), LocScale (Version 0.1)

For manuscripts utilizing custom algorithms or software that are central to the research but not yet described in published literature, software must be made available to editors and reviewers. We strongly encourage code deposition in a community repository (e.g. GitHub). See the Nature Portfolio guidelines for submitting code & software for further information.

Data

Policy information about availability of data

All manuscripts must include a data availability statement. This statement should provide the following information, where applicable:

- Accession codes, unique identifiers, or web links for publicly available datasets
- A description of any restrictions on data availability
- For clinical datasets or third party data, please ensure that the statement adheres to our policy

Source data are provided with this paper. SynDLP cryo-EM map and the corresponding refined atomic model were deposited in the corresponding databank under the following IDs: EMD-14993 and PDB ID-7ZW6.

Human research participants

Policy information about studies involving human research participants and Sex and Gender in Research.

Reporting on sex and gender	N/A
Population characteristics	N/A
Recruitment	N/A
Ethics oversight	N/A

Note that full information on the approval of the study protocol must also be provided in the manuscript.

Field-specific reporting

Please select the one below that is the best fit for your research. If you are not sure, read the appropriate sections before making your selection.

☒ Life sciences ☐ Behavioural & social sciences ☐ Ecological, evolutionary & environmental sciences

For a reference copy of the document with all sections, see nature.com/documents/nr-reporting-summary-flat.pdf

Life sciences study design

All studies must disclose on these points even when the disclosure is negative.

Sample size	No statistical method was used to predetermine the sample size. A sufficient number of micrographs was collected (8,322) to generate a high-resolution reconstruction.
Data exclusions	Micrographs of poor particle coverage and ice quality were discarded.
Replication	The sample size was at least three. Biochemical and biophysical analyses of proteins were reproduced typically using two independent purifications of the recombinant protein and at least three independent replications of each experimental condition. Exception: It was not possible to exactly reproduce the results of the membrane fusion assay measurements as the exact course of the curves highly depends on the actual LUV preparation und the timing of each measurement. However, the trend discussed in the manuscript was always highly reproducible.
Randomization	Randomization is not relevant for cryo-EM data colection, structure analysis or biochemical in vitro assays.
Blinding	Blinding is not relevant for cryo-EM data colection, structure analysis or biochemical in vitro assays.

Reporting for specific materials, systems and methods

We require information from authors about some types of materials, experimental systems and methods used in many studies. Here, indicate whether each material, system or method listed is relevant to your study. If you are not sure if a list item applies to your research, read the appropriate section before selecting a response.

Materials & experimental systems

n/a	Involved in the study
☐	☒ Antibodies
☒	☐ Eukaryotic cell lines
☒	☐ Palaeontology and archaeology
☒	☐ Animals and other organisms
☒	☐ Clinical data
☒	☐ Dual use research of concern

Methods

n/a	Involved in the study
☒	☐ ChIP-seq
☒	☐ Flow cytometry
☒	☐ MRI-based neuroimaging

Antibodies

Antibodies used	α-SynDLP: Polyclonal antibody raised in a rabbit against a truncated SynDLP variant (aa 300 - 812; sequence parts containing conserved GTPase Domain motifs were omitted from the recombinant protein). Produced at Davids Biotechnologie (Regensburg, Germany) solely for application in our Lab. Dilution: 1:2000<br>α-Rabbit-HRP: supplier Sigma-Aldrich Chemie GmbH (Taufkirchen, Germany; catalog number A0545). Dilution: 1:160000
Validation	α-SynDLP was produced for usage in our Lab and validated via Western Blot analyses using the heterologously produced protein.
